# Off-label prescribing of antipsychotics: prescribing practices and clinical experiences of Finnish physicians

**DOI:** 10.1192/j.eurpsy.2022.876

**Published:** 2022-09-01

**Authors:** N. Rautio, L. Ylitolonen, M. Haapea, H. Huovinen, A.-E. Alakokkare, S. Niemelä, J. Miettunen, M. Penttilä, H. Koponen, J. Seppälä, M. Isohanni, E. Jääskeläinen

**Affiliations:** 1 University of Oulu, Center For Life Course Health Research, Oulu, Finland; 2 Oulu University Hospital, Psychiatry, Oulu, Finland; 3 University of Turku, Faculty Of Medicine, Department Of Clinical Medicine, Turku, Finland; 4 University of Helsinki, Faculty Of Medicine, Department Of Psychiatry, Helsinki, Finland

**Keywords:** Antipsychotics, Off-label, physicians, Questionnaire

## Abstract

**Introduction:**

Off-label use of antipsychotics has increased in many countries. In adult populations antipsychotics off-label prescriptions varied from 40 to 75% of all AP users.

**Objectives:**

To examine the off-label prescribing practices and experiences of antipsychotic medication in Finland.

**Methods:**

An electronic questionnaire on physicians’ prescription practices of antipsychotics, especially for off-label use, was sent in 2019 for physicians (n=1195) in different health care facilities including primary health care, occupational health care, in- and outpatient mental health services and services for substance abuse. The sample was selected by systematic and convenience sampling covering five university hospital areas in Finland.

**Results:**

In total, 216 physicians (18% of the target sample) participated in the study, and 94% had prescribed antipsychotics for off-label use. The most common off-label indications were insomnia and anxiety. The most common antipsychotic used was quetiapine. Off-label antipsychotics was not prescribed as a first-choice medication: 99% of the physicians reported that the patients with off-label use have previously had other medications for the corresponding symptoms. In all, 88% of clinicians monitored the patients’ clinical condition, whereas metabolic values were followed more rarely. About 68% of physicians reported more benefit than harm from the antipsychotics off-label use.

**Conclusions:**

Antipsychotics are often prescribed for off-label use, most commonly for insomnia and anxiety. Most of the physicians see more benefits than harms for the patient in off-label use. There is a need to analyse the long-term benefits and harms of off-label use of antipsychotics and create more detailed treatment algorithms and clinical recommendations for such use.

**Disclosure:**

No significant relationships.

